# Multimaterial Microfluidic 3D Printing of Textured Composites with Liquid Inclusions

**DOI:** 10.1002/advs.201800730

**Published:** 2018-11-28

**Authors:** Xiying Li, Jia Ming Zhang, Xin Yi, Zhongyi Huang, Pengyu Lv, Huiling Duan

**Affiliations:** ^1^ State Key Laboratory for Turbulence and Complex Systems Department of Mechanics and Engineering Science BIC‐ESAT College of Engineering Peking University Beijing 100871 China; ^2^ CAPT HEDPS and IFSA Collaborative Innovation Center of MoE Peking University Beijing 100871 China

**Keywords:** composites, direct ink writing, liquid inclusions, microfluidics, multimaterial 3D printing

## Abstract

3D printing with a high degree of spatial and compositional precision could open new avenues to the design and fabrication of functional composites. By combining the direct ink writing and microfluidics, a multimaterial 3D printing system for fabricating textured composites with liquid inclusions of programmable spatial distribution and compositions is reported here. Phase diagrams for the rational selection of desired printing parameters are determined through a combination of simple theoretical analysis and experimental studies. 1D, 2D, and 3D structures programmed with desired inclusion patterns and compositions are fabricated. Moreover, the versatility of this 3D printing framework in fabricating layered composite beams of tunable thermal property and self‐healing materials is demonstrated. The proposed multimaterial microfluidic 3D printing framework could be broadly applicable for structural composites and soft robotic devices.

Natural materials with heterogeneous and hierarchical structures exhibit outstanding mechanical properties, including high specific stiffness, strength, and damage tolerance.^1^, ^2^ For example, dactyl clubs of harlequin mantis shrimps have significantly high specific strength and toughness due to the inorganic ion contents and spatial variation of the mineral phase in the organic matrix, and the graded and layered structures of the dactyl clubs could retard crack propagation.^3^ Similar microstructural variations have also been observed in balsa wood^4^ and bone.^5^ Though many fabrication methods have been proposed to produce biomimetic engineered materials,^6^, ^7^, ^8^, ^9^ composite materials with finely controlled microstructures far exceed the fabrication ability of the conventional manufacturing methods.

Recent developments on the structure design and multiple material printing in additive manufacturing indicate that direct ink writing, an extrusion‐based additive manufacturing technique, can potentially serve as a promising way to fabricate composites with finely controlled microstructures. It has been reported that the direct ink writing utilizing inks of different mechanical and physical properties has been used to fabricate particle laden functional porous materials,^10^, ^11^, ^12^, ^13^, ^14^, ^15^ flexible microelectrodes,^16^ microcapacitors,^17^ electromagnetic structures,^18^ and 3D gradient materials.^19^, ^20^, ^21^ Solid inclusions such as carbon fibers,^22^, ^23^ lead‐free nanowires,^24^ and liquid crystals^25^ dispersed in the inks can be reorientated along the printing direction under the shear and extensional flow in the printhead nozzle. The spatial orientation of the carbon fibers in the epoxy matrix can also be controlled locally by rotating the nozzle during printing.^26^ Moreover, for functionalized fibers, external force fields can be used to control their orientation in the inks. For example, the ultrasonic standing wave is used to control the arrangement of glass fibers in photocurable resin.^27^ Magnetized stiff platelets are orientated in the printed objects by applying low magnetic fields.^28^ Orientational control over carbon nanotubes in the electrical field is used to fabricate biomimetic architectures.^29^ Despite recent advances on the local control over fiber orientation in additive manufacturing, multimaterial 3D printing with a high degree of spatial and compositional precision has not been accomplished, which could open new opportunities for fabricating composite systems of excellent functional properties.^1^, ^2^

Taking advantage of the fine manipulation of droplets in microfluidics,^30^, ^31^, ^32^, ^33^ here we combine direct ink writing and microfluidics to build a multimaterial 3D printing system for fabricating textured composites with liquid inclusions of programmable distribution and compositions. Depending on the geometry of the nozzle, flow rate, printing speed, and spatial selection, structures with different inclusion packing patterns are printed. A combination of simple theoretical analysis and experimental studies is employed to determine quantitative phase diagrams serving as guide in the rational selection of desired printing parameters. Moreover, 1D, 2D, and 3D structures programmed with inclusion packing patterns and compositions are fabricated. Finally, we demonstrate new implications of this microfluidic‐based versatile printing framework in fabricating layered composite beams of tunable thermal expansion property and self‐healing materials. As the liquid inclusions are generated in the form of droplets in the microfluidic part of our printing system, we use droplets and liquid inclusions interchangeably hereinafter except in the discussion on the contact angle measurement of the matrix material.

**Figure** **1** schematically illustrates the multimaterial 3D printing system consisting of a microfluidic part, a 3D printing part, and a custom‐designed single chip microcomputer (SCM). In the microfluidic part, the inner and outer fluids are injected independently at controlled flow rates by syringe pumps (LSP02‐2A, Longer Precision Pump) into the inlet channels and meet at the T‐junction in a generator of on‐demand mixing droplets which is integrated to produce liquid inclusions of simple and mixed compositions in printed structures (see Figure S1, Supporting Information). In the droplet generator, two inner phases (red and blue) are mixed first to produce (purple) mixed output which is then sheared into droplets by the continuous immiscible outer phase at the T‐junction. Depending on the flow rate ratio between the red and blue inner phases, the output droplets can change their color (indicating the degree of mixing) from red to purple, then to blue in a continuous manner. In our operation windows of the inner phase flow rate *Q*_i_ and outer phase flow rate *Q*_o_, the droplets (serving as liquid inclusions in printed composites) are formed in a dripping regime to ensure their monodispersity during the printing process. As the pumping of each inner phase is controlled independently, connecting the droplet generator with a dual‐nozzle printhead enables us to print composites with two kinds of liquid inclusions simultaneously. As demonstrated in later discussions, such a printing approach has been used in our fabrication of self‐healing polymer composites in which two‐part curing agents are encapsulated separately in two different kinds of liquid inclusions and enable a rebinding of the crack face with their release in the damage region.

**Figure 1:**
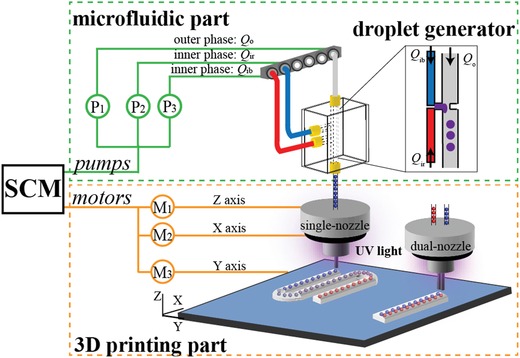
Schematic of the multimaterial microfluidic 3D printing platform for generating composites with liquid inclusions. The printing platform consists of a microfluidic part for generating inks containing liquid inclusions, a 3D printing part for the structure printing, and a single chip microcomputer (SCM) for the cooperative control of the fluid injection in the microfluidic part, the motion of the printing stage along *Y*‐axis, and the nozzle motion along *X*‐ and *Z*‐axes. Arrows in the microfluidic part represent the flow direction.

Water‐in‐resin two phase fluids are exemplified as the ink in our printing demonstrations, where the inner phase is the water–glycerol mixture and the outer phase is the commercial photosensitive liquid resin which can be regarded as a Newtonian fluid before it is cured (Figure S2, Supporting Information). In the dripping regime, the droplet diameter *D*_drop_ scales as $D_{\mathrm{drop}}\sim w_{o}Ca_{o}^{-1}$ (Figure S3, Supporting Information),^30^ where *w*_o_ is the outlet channel width and the outer phase capillary number *Ca*_o_ = η_o_ *u*_o_/γ denotes the relative strength between the viscous force and interfacial tension. Here η_o_ (= 0.53 Pa s) and *u*_o_ represent the outer phase viscosity and flow velocity at the T‐junction, respectively, and γ (= 0.02 N m^−1^) is the interfacial tension between the inner and outer phases. In our system, the outlet channel width is *w*_o_ = 330 µm, and the diameter of the embedded droplets ranges from 200 to 500 µm (Figure S3, Supporting Information). During the printing process, droplets in the gentle flow (*Ca*_o_ < 0.1) could pass through the printhead nozzle without breaking due to a high degree of the droplet deformability.^34^

We first benchmark printing on the glass substrate 1D composite lines and 2D carpet‐like structures consisting of water droplets embedded in the resin matrix. During the printing of 1D lines, the nozzle of a diameter *D*_nozzle_ = 0.33 mm moves at a tip height *H* and a printing speed *v*. The contact angle of resin on the glass substrate is 38° ± 1° at 25 °C. As the height of the printed line is less than the characteristic capillary length (1.4 mm), the gravity effects can be ignored and the printed line adopts a cylindrical cap shape of width *w*, contact angle θ, and height *h* = *w*(1 − cos θ)/(2sin θ), and its cross‐sectional area is $A=w^{2}(\theta {\mathrm{/sin}}^{2}\theta -\cot\theta )/4$. At a total flow rate *Q* = *Q*_i_ + *Q*_o_, *A* = *Q*/*v*. Therefore, we have $w=2\sqrt{Q/v}/\sqrt{\theta {\mathrm{/sin}}^{2}\theta -\cot\theta }$. This linear relationship between *w* and $\sqrt{Q/v}$ is confirmed by the experimental results (Figure S4a, Supporting Information). At a large tip height *H* > *h*, the extruded ink could form a droplet hanging at the nozzle tip and lose contact with the printed line segment as the nozzle moves. Therefore, a discontinuous 1D line with multiple segments (regime I in **Figure** **2**a) is printed as *h* < *H*, and the corresponding criterion is *H* > *w*(1 − cos θ)/(2sin θ) or

(1) $$\frac{H}{D_{\mathrm{nozzle}}}>\frac{1-\cos\theta }{\sqrt{2\theta -\sin2\theta }}\sqrt{\frac{\pi c}{2v}}$$

where $c=Q/(\pi D_{\mathrm{nozzle}}^{2}/4)$ is the extrusion speed of the composite ink from the nozzle.

**Figure 2:**
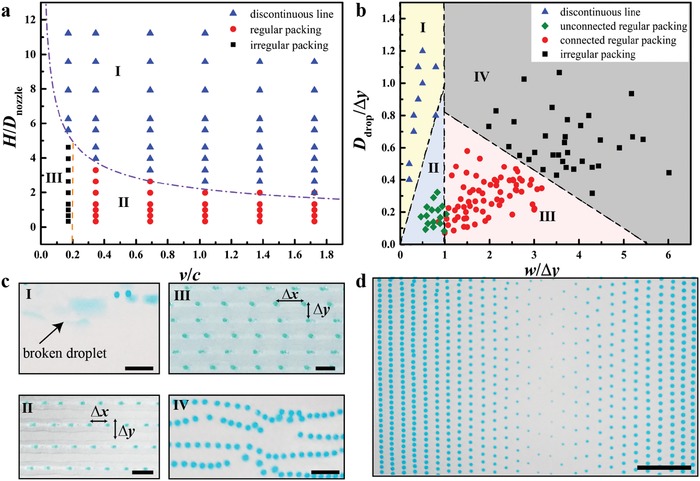
Benchmark printing of the 1D lines and 2D carpet‐like structures. a) The phase diagram of the droplet packing patterns in printed 1D lines. Symbols are the experimental results and phase boundaries are predicted theoretically by Equations (1) and (2). b) The phase diagram of the droplet packing in terms of the normalized droplet diameter *D*_drop_/Δ*y* and line width *w*/Δ*y* for the printed 2D carpet‐like structures. c) Experimental illustrations of the droplet packing phases in (b). d) Printed 2D sample with programmed inclusion spacing and sizes. Scale bars represent 2 mm in (c) and 5 mm in (d). Printing parameters can be found in the Experimental Section.

At *D*_drop_ < *w*, we have *V*_droplet_/*V*_resin_ = *Q*_i_/*Q*_o_. As *V*_resin_ = *A*· Δ*x* − *V*_droplet_, the droplet spacing Δ*x* along the printed line is $\Delta x=(\pi D_{\mathrm{drop}}^{3}v)/(6Q_{i})$, consistent with the experimental results (Figure S4b, Supporting Information). In the case of Δ*x* < *D*_drop_, the droplets contact or overlap and form an irregular packing configuration with merged inclusions in the printed line. The criterion for the irregular inclusion packing (regime III in Figure 2a) is $\Delta x=(\pi D_{\mathrm{drop}}^{3}v)/(6Q_{i})<D_{\mathrm{drop}}$ or

(2) $$\frac{v}{c_{i}}<\frac{3}{2}\frac{D_{\mathrm{nozzle}}^{2}}{D_{\mathrm{drop}}^{2}}$$

where $c_{i}=Q_{i}/(\pi D_{\mathrm{nozzle}}^{2}/4)$ is the extrusion speed of the inner phase.

In the rest regime of Δ*x* > *D*_drop_ and *H* < *h* (regime II in Figure 2a), the line with regularly packed liquid inclusions is printed as theoretically predicted.

In the fabrication of a 2D carpet‐like structures consisting of liquid inclusions encapsulated in the resin matrix, we program parallel straight paths of the nozzle motion at *H* = 150 µm and conduct printing in the regime II in Figure 2a (regular packing regime). In addition to the parameters of line width *w* and droplet diameter *D*_drop_ as discussed in 1D line printing, a new length parameter, the distance Δ*y* between two adjacent printing paths, emerges and regulates the printing of 2D structures. In the cases of Δ*y* > *w*, the printed lines do not overlap and stand alone. In these circumstances, the droplet of diameter *D*_drop_ > *w* would form individual clusters and a discontinuous line is printed along each printing path (regime I in Figure 2b); for the droplet of diameter *D*_drop_ < *w*, the droplets are regularly packed in the printed lines standing alone (unconnected regular packing regime II arising, see Figure 2b). Note that the droplet inclusions in the discontinuous line in Figure 2a remain intact, while these in discontinuous line in Figure 2b break up.

In the cases of Δ*y* < *w*, the printed lines in adjacent printing paths would form contact with each other and a resin meniscus forms attaching to the nozzle tip and moves during the printing process. Experimental observation indicates that the characteristic size of the meniscus is around *l ≈* 0.18*w*. In these cases, droplets of *D*_drop_ > Δ*y* might overlap and an irregular packing arises; at *D*_drop_ < Δ*y* < *D*_drop_ + *l*, the moving meniscus near the nozzle induces an irregular motion of the existing droplets and an irregular packing pattern forms. In both cases, we can observe the regime IV in Figure 2b.

In the rest regime of the *D*/Δ*y − w/*Δ*y* space (*w*/Δ*y* > 1 and *D*/Δ*y* < 1 − 0.18*w*/Δ*y*), the printed lines contact and a 2D carpet‐like structure is formed with droplets dispersed in a regular pattern (connected regular packing, regime III in Figure 2b).

Based on the analyses above, a packing phase diagram in 2D printing is determined and confirmed experimentally (Figure 2b) and the corresponding printed structures can be found in Figure 2c. The benchmark printing above with corresponding simple analyses show that the liquid inclusion arrangement in the printed objects could be precisely programmed. To validate this in situ control, we print a structure with programmed droplet size and spacing by simply changing the inner phase flow rate (Figure 2d). Decreasing the inner phase flow rate results in a decreased droplet size and an increased droplet spacing at a given printing speed.

In the above experiments, the density of the droplets is 1.138 kg cm^−3^, and the resin matrix has a density of 0.98 kg cm^−3^ and a viscosity of 530 mPa s at 26 °C. Due to the small density difference between the droplets and resin matrix and the high viscosity of the resin matrix, the embedded droplets are stable and could remain intact even as the printed line is lifted and deformed (Figure S5b, Supporting Information). In the case where the density difference between the inclusions and the fluidic matrix is significantly different, the inclusions of a smaller density rise to the matrix surface while the inclusions of a larger density could sink. As indicated in our additional experiment (Figure S5c, Supporting Information), the bubble inclusions initially in the resin matrix rise due to their much smaller density and then break at the resin surface.

The in situ programmable generation of liquid inclusions in the matrix material offers the ability to print more complex structures with locally tunable droplet composition and distribution. To demonstrate these abilities, we print 1D, 2D, and 3D objects containing liquid inclusions. In **Figure** **3**a and Video S1 (Supporting Information), we fabricate a spiral structure with droplet inclusions evenly spaced along the structure curve. In a programmed continuous printing, we fabricate a 2D structure with droplet inclusions in a pattern showing the letters PKU (Figure 3b and Video S2, Supporting Information) and with droplets of colors gradually varying from red to blue (Figure 3c and Video S3, Supporting Information). The total flow rate of the red and blue inner phases is fixed to obtain evenly spaced droplets at a constant printing speed. We program the distribution of the liquid inclusions in printing 3D objects. In the textured object in Figure 3d, the droplet spacing is precisely controlled in directions parallel and perpendicular to the printing path. This scheme of fine control on the droplet spacing could be modified for display applications. In Figure 3e, a quadrangle honeycomb architecture with monodisperse liquid inclusions is printed to demonstrate the ability of precise control over the inclusion spatial distribution. A printed block with three colors demonstrates the fast responses to the flow rate changing as well as to the mixing in red and blue inner phases (Figure 3f). In addition to printing structures with controlled inclusion distribution and composition with fast response time, this printing approach simplifies the preparation of composite inks and reduces the amount of waste material by direct dispersing inclusion materials at specified volumes.

**Figure 3:**
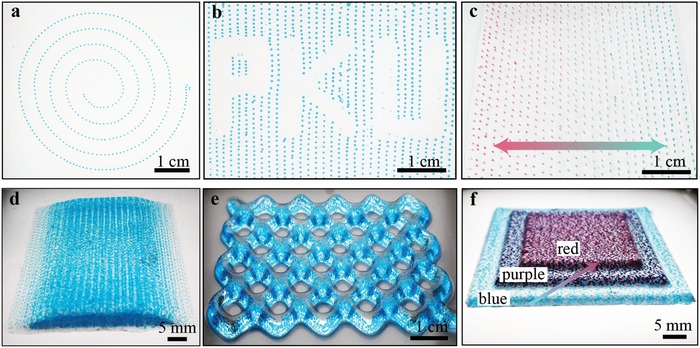
1D, 2D, and 3D structures printed by the multimaterial microfluidic 3D printing system. a) 1D spiral line with evenly spaced liquid inclusions. b) 2D layer with droplet inclusions in a pattern showing the letters PKU. c) 2D layer containing droplets of deliberately controlled compositions indicated by color gradient. d) Trapezoid block and e) 3D quadrangle honeycomb architecture with monodisperse liquid inclusions. f) Block with three steps, each consisting of inclusions filled with liquid of a specified color.

To illustrate the potential of this multimaterial microfluidic 3D printing technique in producing functional composites, we fabricate a beam device which could undergo programmed shape changes when triggered by a thermal stimulus and print a self‐healing polymer composite containing liquid inclusions filled with healing agents.

In the example of the beam device, we fabricate a two‐layer beam with a resin top layer and a bottom layer consisting of liquid inclusions embedded in a resin matrix. As the thermal expansion properties of the liquid inclusions and the resin material are different, the top and bottom layers would differ in length due to the thermal mismatch strain and the beam bends. The effective bulk modulus κ_e_ and thermal expansion coefficient α_e_ of the bottom composite layer are given by the micromechanics theory as^35^, ^36^

(3) $$\kappa_{e}=\frac{3\kappa_{m}\kappa_{i}+4\mu_{m}[(1-c_{i})\kappa_{m}+c_{i}\kappa_{p}]}{3[(1-c_{i})\kappa_{i}+c_{i}\kappa_{m}]+4\mu_{m}}$$

(4) $$\frac{\alpha_{i}\kappa_{i}-\alpha_{m}\kappa_{m}}{\kappa_{i}-\kappa_{m}}=\frac{\alpha_{e}\kappa_{e}-[c_{i}\alpha_{i}\kappa_{i}+(1-c_{i})\alpha_{m}\kappa_{m}]}{\kappa_{e}-[c_{i}\kappa_{i}+(1-c_{i})\kappa_{m}]}$$

where κ, *µ*, and α represent the bulk modulus, shear modulus, and thermal expansion coefficient, respectively. *c*_i_ is the volume fraction of the liquid inclusions. Subscripts “i” and “m” are used to identify quantities associated with the liquid inclusions and matrix, respectively. Here we have assumed that the shear modulus of the liquid inclusions is negligible. The mechanical properties of the resin matrix are *µ*_m_ = 0.36 GPa and κ_m_ = 2.56 GPa, and κ_i_ = 2.20 GPa for the water–glycerol inclusions. At *c*_i_ = 15%, the effective bulk modulus is determined as κ_e_ = 1.70 GPa and the effective Young's modulus is predicted as *E*_e_ = 2(1 − 2ν)κ_e_ = 1.00 GPa with Poisson ratio *ν =* 0.40. As shown in **Figure** **4**a, the theoretical prediction of *E*_e_ agrees well with the experimental result 0.96 GPa derived from the stress–strain curve in uniaxial tensile test. Thermal expansion tests indicate that the thermal expansion coefficients of the resin matrix and the composite (*c*_i_ = 15%) vary with temperature nonmonotonically (Figure 4b). Negative thermal expansion coefficients near 70 °C for both the resin matrix and the composite might be due to the glass transition of the resin. With the knowledge of the effective Young's modulus and thermal expansion coefficient of the composite, the thermal and mechanical behavior of the two‐layer beam with a resin top layer and a bottom layer consisting of liquid inclusions embedded in a resin matrix can be determined and predicted. Here we perform experimental and theoretical analysis on such a two‐layer beam fabricated using our microfluidic 3D printing system. The thickness of each layer in the printed two‐layer beam is *H* = 150 µm. Due to the different thermal expansion properties, the beam upon a temperature rise from *T*_0_ = 26 °C to *T*_1_ = 100 °C would bend into a circular arc of radius *R* as estimated by the beam theory on the thermal deflection of bimetal strip thermostat^37^ (Supporting Information) and is given by

(5) $$\frac{R}{H}=\frac{14+\frac{E_{m}}{E_{e}}+\frac{E_{e}}{E_{m}}}{12\int_{T_{0}}^{T_{1}}(\alpha_{e}-\alpha_{m})dT}$$

where α_m_ and α_e_ are the thermal expansion coefficients of the resin top layer and the composite bottom layer, respectively. Taking their values in Figure 4b, *R*/*H* in Equation (5) is estimated as 500 at 100 °C. Then we have *R* = 7.5 cm. The theoretical prediction (yellow dashed line in Figure 4c) agrees well with the experimental result. In Figure 4a–c, the volume fraction of liquid inclusions in the composite layer is *c*_i_ = 15%. To investigate how the mechanical behaviors of the beam depend on *c*_i_ in the bottom layer, we fabricate two‐layer beams of different *c*_i_ and investigate their thermal deflection. As shown in Figure 4d, the two‐layer beam exhibits larger deflection as *c*_i_ increases which means that the effective thermal expansion coefficient of the composite bottom layer increases as *c*_i_ increases. This is consistent with our theoretical prediction in Figure S6 (Supporting Information). As a featured demonstration, a smart structure with eight two‐layer beams, which can switch reversibly between the flat and curved configurations upon thermal stimulus, is designed and fabricated (see Figure 4e). Taking advantage of the shape memory ability of the resin materials, the structure in the curved configuration could recover its original flat configuration upon slow cooling, and maintain the current configuration upon a fast cooling which can then undergo configuration recovery when reheated and slowly cooled. Such a smart structure has immediate potential applications in self‐folding structures, structural composites, and soft actuator–based robots and devices.^38^, ^39^

**Figure 4:**
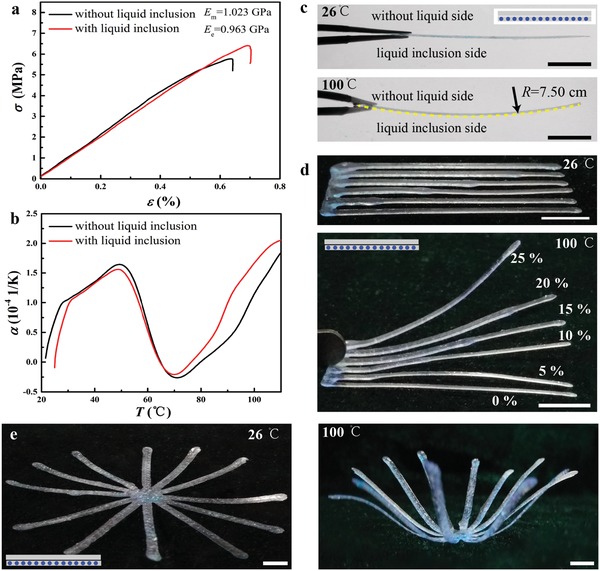
Thermomechanical behaviors of printed composites made up of resin matrix with water–glycerol inclusions. a) The uniaxial tensile and b) thermal expansion tests of pure resin and resin‐based composite with liquid inclusions at a volume fraction *c*_i_ = 15%. c) Thermal deflection of a printed two‐layer beam (top layer, pure resin; bottom composite layer, resin containing liquid inclusions). The dashed line shows the theoretical prediction in Equation (5) with *c*_i_ = 15%. d) Bending of two‐layer beams at different *c*_i_ from 0% to 25%. e) Thermally induced flower‐shaped complex based on the two‐layer beam structure. The beam could maintain at the flower‐shaped configuration upon fast cooling to the room temperature. Scale bars, 1 cm.

As a final demonstration on the versatility of this microfluidic‐based 3D printing system, we fabricate a self‐healing polymer composite consisting of liquid inclusions filled with two‐part epoxy adhesive healing agents (Devcon, No. 14270) using the dual‐nozzle printhead (**Figure** **5**). Healing agents A (epoxy resin) and B (curing agent) are dyed red and blue, respectively, and are dispersed as liquid inclusions of the same size in the resin matrix. As the damage is inflicted on the composite material, the healing agents are released by the cracks crossing and fracturing them, and mixed at a volume ratio around 1:1. Within around 5 min local cracks are healed through mending reaction at the damage sites as demonstrated in Figure 5b,c, where a small crack of width around 58 µm and a relatively large crack of width around 500 µm are healed. Further tests indicate that the healed sample could carry an object of weight 200 g, showing the restoration of its mechanical strength (Figure 5c).

**Figure 5:**
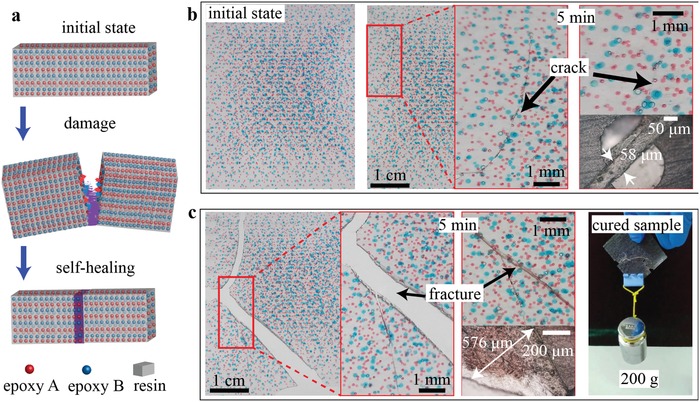
Self‐healing composites printed by the microfluidic 3D printing platform. a) Schematic of the self‐healing process. The damage triggers the material rupture and release of the healing agents (epoxy adhesives A and B here) which then flow toward the damage sites and cause the local mending reaction to heal the crack. b) Self‐healing of small cracks with a width around 58 µm. c) Self‐healing of relatively large cracks with a width around 500 µm. The healed sample is able to carry a weight of 200 g.

In conclusion, we have presented a multimaterial microfluidic 3D printing framework for fabricating textured composites with liquid inclusions of programmable distribution and compositions. Quantitative phase diagrams providing rational guide for the selection of desired printing parameters are determined. Using this microfluidic‐based multimaterial printing framework, 1D, 2D, and 3D structures programmed with inclusion packing patterns and compositions are fabricated. We have also demonstrated the versatility of this 3D printing framework in the implications of producing layered composite beams and self‐healing materials. The presented printing framework demonstrated using resin materials could be applied to a wide range of polymer materials and paves the way for the design and programming of composite material systems.

## Experimental Section

*Microfluidic Chip Fabrication*: The microfluidic chips were designed using Solidworks (Dassault Systèmes) and fabricated with the aid of a stereolithography 3D printer (Form‐2, Formlabs). The printed microfluidic chips were washed by isopropyl alcohol to remove uncured resin, then postcured for 1 h upon UV light (36 W), and eventually polished by fine sandpapers to achieve desired optical transparency.

*Printing System Setup*: The printing system (see Figure 1) is based on a commercial printer LULZBOT (Aleph Objects) with its original printhead replaced by the customized printhead fabricated using a commercial 3D printing system (Objet350 Connex3, Stratasys). Microfluidic chips and the print nozzle were assembled and added to the printhead. The cooperation of the ink pumping and the movement of the nozzle and the printing stage was achieved using the Arduino board (Zduino UNO) and Rambo board (Rambo rev 1.3 L). Commercial photosensitive resin (CLEAR PLGPCL04, Formlabs, density 0.98 kg cm^−3^) was used as the outer phase, and water–glycerol mixture (volume fraction 1:1, density 1.138 kg cm^−3^, viscosity 6.9 mPa s at 26 °C) dyed by colorant was used as the inner phase in a microfluidic flow. In the microfluidic chips, these two phases formed the ink consisting of water–glycerol inclusions embedded in the resin phase. The ink was extruded out of the blunt nozzle tip and was cured under a UV lamp (light wavelength 395 nm, output power 8 W) installed on the printhead.

*Contact Angle Measurement*: Pure resin droplets, each with a volume of 0.3 µL, were deposited by a syringe on the glass substrate. The instrument Contact Angle Meter (Data Physics Corp.) was used to measure the resin contact angle. Data for each droplet were expressed as an average of five measurements.

*Line Printing*: In the printing of 1D lines (Figure 2a), the nozzle moved at different tip heights *H* from 170 to 500 µm and five different printing speeds *v* from 120 to 600 mm min^−1^. The inner and outer phase flow rates *Q*_i_ and *Q*_o_ were fixed as 5 and 25 µL min^−1^, respectively. The liquid inclusion sizes and spacing were analyzed by an image processing program ImageJ.

*Printing of 2D Carpet‐Like Structure*: The nozzle height was fixed at 150 µm and the same printing speeds as those in Figure 2a are used. The spacing between the parallel straight nozzle motion paths from left to right in Figure 2b are 0.5, 0.7, 1.0, 1.2, 1.5, 1.8, 2.0, 2.5, and 3.0 in the units of mm. The inner phase flow rates *Q*_i_ were taken at 5, 5, 5, 5, 8, 6, 6, and 4, and the corresponding outer phase flow rates *Q*_o_ were 5, 10, 15, 20, 18, 14, 20, and 6. All flow rates were in the units of µL min^−1^. Inclusion sizes and spacing were analyzed by ImageJ.

*Mechanical and Thermal Testing*: Tension tests of the printed samples were performed by the mechanical testing system MTI Instruments (MTESTQuattro, ADMET Inc.). The thermal expansion coefficients were determined using the thermal testing device Dilatometer (DIL402C).

## Conflict of Interest

The authors declare no conflict of interest.

## Supporting information

Supplementary

Supplementary

Supplementary

Supplementary

## References

[1] J. W. C. Dunlop , P. Fratzl , Annu. Rev. Mater. Res. 2010, 40, 1.

[2] R. Wang , H. S. Gupta , Annu. Rev. Mater. Res. 2011, 41, 41.

[3] U. G. K. Wegst , H. Bai , E. Saiz , A. P. Tomsia , R. O. Ritchie , Nat. Mater. 2015, 14, 23. 25344782 10.1038/nmat4089

[4] A. D. Silva , S. Kyriakides , Int. J. Solids Struct. 2007, 44, 8685.

[5] P. Fratzl , Nat. Mater. 2008, 7, 610. 18654582 10.1038/nmat2240

[6] A. Sellinger , P. M. Weiss , A. Nguyen , Y. Lu , R. A. Assink , W. Gong , C. J. Brinker , Nature 1998, 394, 256.

[7] F. Bouville , E. Maire , S. Meille , B. Van de Moortele , A. J. Steveson , S. Deville , Nat. Mater. 2014, 13, 508. 24658117 10.1038/nmat3915

[8] C. Hao , Y. Liu , X. Chen , J. Li , M. Zhang , Y. Zhao , Z. Wang , Small 2016, 12, 1825. 26865317 10.1002/smll.201503060

[9] X. Chen , J. Wu , R. Ma , M. Hua , N. Koratkar , S. Yao , Z. Wang , Adv. Funct. Mater. 2011, 21, 4617.

[10] M. R. Sommer , M. Schaffner , D. Carnelli , A. R. Studart , ACS Appl. Mater. Interfaces 2016, 8, 34677. 27933765 10.1021/acsami.6b11440

[11] C. Minas , D. Carnelli , E. Tervoort , A. R. Studart , Adv. Mater. 2016, 28, 9993. 27677912 10.1002/adma.201603390

[12] J. T. Muth , P. G. Dixon , L. Woish , L. J. Gibson , J. A. Lewis , Proc. Natl. Acad. Sci. USA 2017, 114, 1832. 28179570 10.1073/pnas.1616769114 PMC5338428

[13] T. Yang , Y. Hu , C. Wang , B. P. Binks , ACS Appl. Mater. Interfaces 2017, 9, 22950. 28636315 10.1021/acsami.7b05012

[14] M. R. Sommer , L. Alison , C. Minas , E. Tervoort , P. A. Ruhs , A. R. Studart , Soft Matter 2017, 13, 1794. 28165099 10.1039/c6sm02682f

[15] B. An , Y. Ma , W. Li , M. Su , F. Li , Y. Song , Chem. Commun. 2016, 52, 10948. 10.1039/c6cc05910d 27531042

[16] B. Y. Ahn , E. B. Duoss , M. J. Motala , X. Guo , S.‐I. Park , Y. Xiong , J. Yoon , R. G. Nuzzo , J. A. Rogers , J. A. Lewis , Science 2009, 323, 1590. 19213878 10.1126/science.1168375

[17] W. Li , Y. Li , M. Su , B. An , J. Liu , D. Su , L. Li , F. Li , Y. Song , J. Mater. Chem. A 2017, 5, 16281.

[18] N. Zhou , C. Liu , J. A. Lewis , D. Ham , Adv. Mater. 2017, 29, 1605198. 10.1002/adma.201605198 28198059

[19] J. O. Hardin , T. J. Ober , A. D. Valentine , J. A. Lewis , Adv. Mater. 2015, 27, 3278. 10.1002/adma.201500222 25885762

[20] W. Liu , Y. S. Zhang , M. A. Heinrich , F. De Ferrari , H. L. Jang , S. M. Bakht , M. M. Alvarez , H. Yang , Y.‐C. Li , G. T. de Santiago , A. K. Miri , K. Zhu , P. Khoshakhlagh , H. Cheng , X. Guan , Z. Zhong , J. Ju , G. H. Zhu , X. Jin , S. R. Shin , M. R. Dokmeci , A. Khademhosseini , Adv. Mater. 2017, 29, 1604630. 10.1002/adma.201604630 PMC5235978 27859710

[21] T. J. Ober , D. Foresti , J. A. Lewis , Proc. Natl. Acad. Sci. USA 2015, 112, 12293. 26396254 10.1073/pnas.1509224112 PMC4603479

[22] B. G. Compton , J. A. Lewis , Adv. Mater. 2014, 26, 5930. 24942232 10.1002/adma.201401804

[23] R. Matsuzaki , M. Ueda , M. Namiki , T.‐K. Jeong , H. Asahara , K. Horiguchi , T. Nakamura , A. Todoroki , Y. Hirano , Sci. Rep. 2016, 6, 23058. 26965201 10.1038/srep23058 PMC4786850

[24] M. Gao , L. Li , W. Li , H. Zhou , Y. Song , Adv. Sci. 2016, 3, 1600120. 10.1002/advs.201600120 PMC5089621 27840806

[25] A. Kotikian , R. L. Truby , J. W. Boley , T. J. White , J. A. Lewis , Adv. Mater. 2018, 30, 1706164. 10.1002/adma.201706164 29334165

[26] J. R. Raney , B. G. Compton , J. Mueller , T. J. Ober , K. Shea , J. A. Lewis , Proc. Natl. Acad. Sci. USA 2018, 115, 1198. 29348206 10.1073/pnas.1715157115 PMC5819411

[27] T. M. Llewellyn‐Jones , B. W. Drinkwater , R. S. Trask , Smart Mater. Struct. 2016, 25, 02LT01.

[28] D. Kokkinis , M. Schaffner , A. R. Studart , Nat. Commun. 2015, 6, 8643. 26494528 10.1038/ncomms9643 PMC4639895

[29] Y. Yang , Z. Chen , X. Song , Z. Zhang , J. Zhang , K. K. Shung , Q. Zhou , Y. Chen , Adv. Mater. 2017, 29, 1605750. 10.1002/adma.201605750 PMC7032659 28185341

[30] P. Zhu , L. Wang , Lab Chip 2017, 17, 34. 10.1039/c6lc01018k 27841886

[31] S. Ma , N. Mukherjee , E. Mikhailova , H. Bayley , Adv. Biosys. 2017, 1, 8. 10.1002/adbi.201700075 32646178

[32] R. H. Cole , S.‐Y. Tang , C. A. Siltanen , P. Shahi , J. Q. Zhang , S. Poust , Z. J. Gartner , A. R. Abate , Proc. Natl. Acad. Sci. USA 2017, 114, 8728. 28760972 10.1073/pnas.1704020114 PMC5565430

[33] C. W. Visser , T. Kamperman , L. P. Karbaat , D. Lohse , M. Karperien , Sci. Adv. 2018, 4, eaao1175. 29399628 10.1126/sciadv.aao1175 PMC5792224

[34] C. D. Han , K. Funatsu , J. Rheol. 1978, 22, 113.

[35] Z. Hashin , J. Appl. Mech. 1962, 29, 143.

[36] V. M. Levin , Mech. Solids 1967, 2, 58.

[37] S. Timoshenko , J. Opt. Soc. Am. 1925, 11, 233.

[38] H. Lee , C. Xia , N. X. Fang , Soft Matter 2010, 6, 4342.

[39] Q. Ge , A. H. Sakhaei , H. Lee , C. K. Dunn , N. X. Fang , M. L. Dunn , Sci. Rep. 2016, 6, 31110. 27499417 10.1038/srep31110 PMC4976324

