# Supplementary material for: Multimaterial Microfluidic 3D Printing of Textured Composites with Liquid Inclusions
Source: Adv Sci (Weinh). 2018 Nov 28;6(3):1800730. doi: 10.1002/advs.201800730 (PMC6364488; doi:10.1002/advs.201800730)
Supplement: Supplementary file 1 — Supplementary [file ADVS-6-1800730-s002.pdf]

Copyright WILEY-VCH Verlag GmbH & Co. KGaA, 69469 Weinheim, Germany, 2018.

ADVANCED SCIENCE
Open Access

Supporting Information

for *Adv. Sci.,* DOI: 10.1002/advs.201800730

Multimaterial Microfluidic 3D Printing of Textured Composites with Liquid Inclusions

Xiying Li, Jia Ming Zhang, Xin Yi,* Zhongyi Huang, Pengyu Lv, and Huiling Duan*

Supporting Information

Multimaterial microfluidic 3D printing of textured composites with liquid inclusions

Xiying Li, Jia Ming Zhang*, Xin Yi*, Zhongyi Huang, Pengyu Lv and Huiling Duan

Emails: zhangjmedu@163.com; xyi@pku.edu.cn

Contents

Thermal deflection of the printed two-layer beam.
Figure S1 T-junction droplet generator.
Figure S2 Rheological properties of the resin outer phase.
Figure S3 The relationship between the normalized droplet diameter D_{drop}/w_o and the outer phase capillary number Ca_o at the T-junction in the generator of mixing droplets.
Figure S4 Structures of the printed line.
Figure S5 Inclusion positioning induced by the density difference between the inclusions and the fluidic matrix (resin here).
Figure S6 The effective Young's modulus E_e and thermal expansion coefficient α_e.
Figure S7 Thermal deflection of the two-layer beam in a temperature rise.
Video S1 Printing a spiral structure.
Video S2 Printing the letters PKU (abbreviation of Peking University).
Video S3 Producing on-demand mixing droplets.

For the two-layer beam upon thermal deflection in Figure 4 in the main text, the top layer is composed of pure resin and the composite bottom layer consists of liquid inclusions suspended in the resin matrix as illustrated in Figure S7. Each layer has a thickness of H. The thermal expansion coefficient of the bottom layer is larger than that of the top layer as shown in Figure 4b. The initial straight beam has a length L_0 at the room temperature T_0. An increase in temperature to T_1 induces a relative length increase $\Delta L_m = L_0\int_{T_0}^{T_1}\alpha_m dT$ in the bottom and a relative length increase $\Delta L_e = L_0\int_{T_0}^{T_1}\alpha_e dT$ in the top layer, and the beam deforms into a circular arc of an arc angle θ and a radius of curvature R. Here α_m and α_e are the thermal expansion coefficients of the resin top layer and the composite bottom layer, respectively. Based on the beam theory on the thermal deflection of a bi-metal strip termostat, [37] the normalized relative length increase of the interface between the top and bottom layers can be given as $\varepsilon_{it} = \int_{T_0}^{T_1}\alpha_m dT + \frac{H}{12R}\frac{E_m+E_e}{E_m} + \frac{H}{2R}$ or $\varepsilon_{ib} = \int_{T_0}^{T_1}\alpha_m dT - \frac{H}{12R}\frac{E_m+E_e}{E_e} - \frac{H}{2R}$. Then we have $\varepsilon_{it} = \varepsilon_{ib}$ or

$$\frac{R}{H} = \frac{14+\frac{E_m}{E_e}+\frac{E_e}{E_m}}{12\int_{T_0}^{T_1}(\alpha_e-\alpha_m)dT}.$$

Based on the experimental data on E_m and E_e in Figure 4a and α_m and α_e in Figure 4b, R/H is then estimated as 500. As H=150 μm, we have R=7.5 cm which agrees well with the experimental result in Figure 4c. The thickness variation of each layer during the thermal deformation has been ignored.

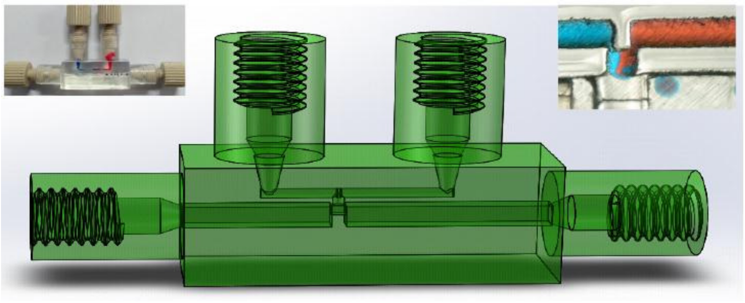

Figure S1. T-junction droplet generator. The upper left inset shows the assembled chip in the printing platform and the upper right inset indicates the on-demand mixing droplet formation.

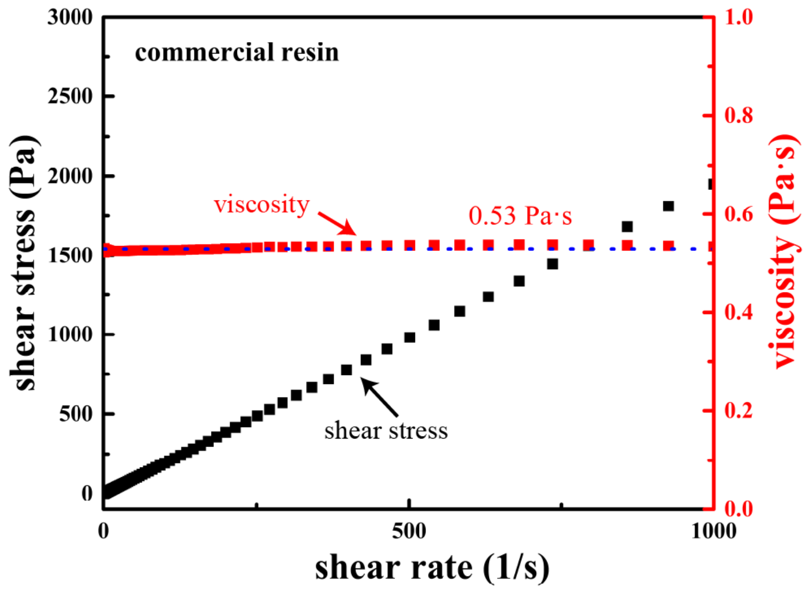

Figure S2. Rheological properties of the resin outer phase.

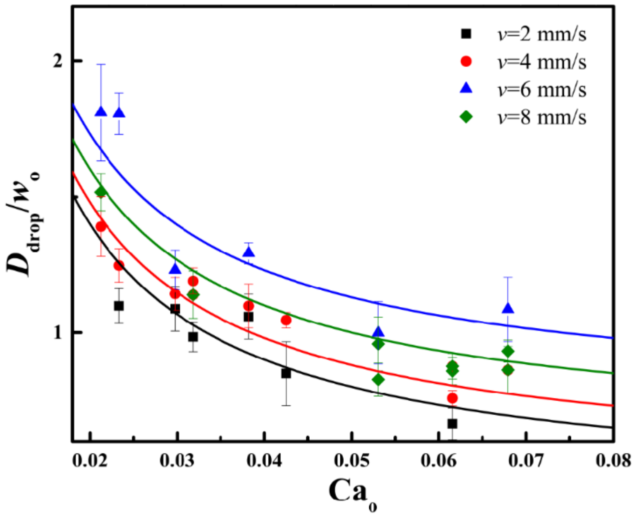

Figure S3. The relationship between the normalized droplet diameter D_{drop}/w_o and the outer phase capillary number Ca_o at the T-junction in the generator of mixing droplets. Symbols represent the experimental data and solid lines show the scaling law $D_{drop}/w_o \sim Ca_o^{-1}$.

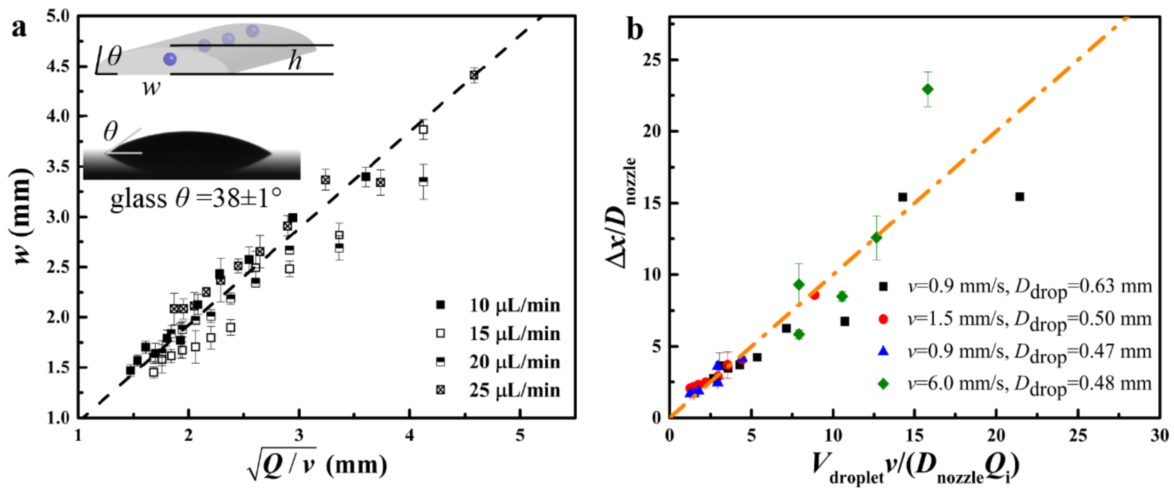

Figure S4. Structures of the printed line. (a) The line width w as a function of $\sqrt{Q/v}$ with Q as the total flow rate and v as the printing speed. Upper inset: schematic of a printed line of width w, height h, and contact angle θ. Lower inset: contact angle of the resin on the glass substrate at room temperature. (b) The linear relationship between the normalized droplet spacing $\Delta x/D_{nozzle}$ and the dimensionless parameter $V_{droplet}v/(D_{nozzle}Q_i)$ with $V_{droplet}=\pi D_{drop}^3/6$. Dots in (a) and (b) are the experimental results and dashed lines represent the theoretical prediction.

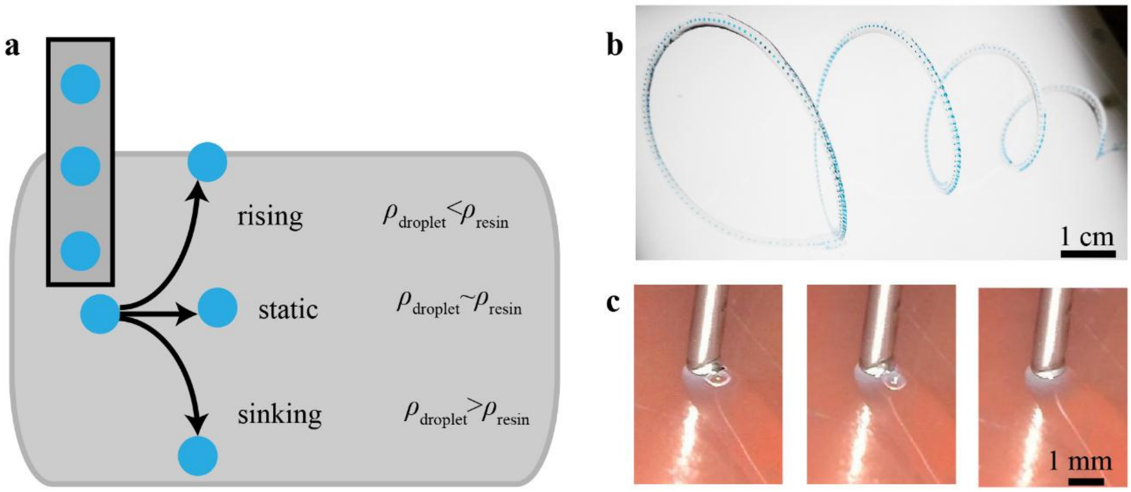

Figure S5. Inclusion positioning induced by the density difference between the inclusions and the fluidic matrix (resin here). (a) Illustration of the positioning of the droplet inclusions. (b) Droplets are stable and remain intact in a helical structure formed by pulling the 1D spiral line in Figure 3a in the main text. (c) In the case of the bubble inclusions, the bubbles of a much smaller density than the matrix first rise and then break at the resin surface.

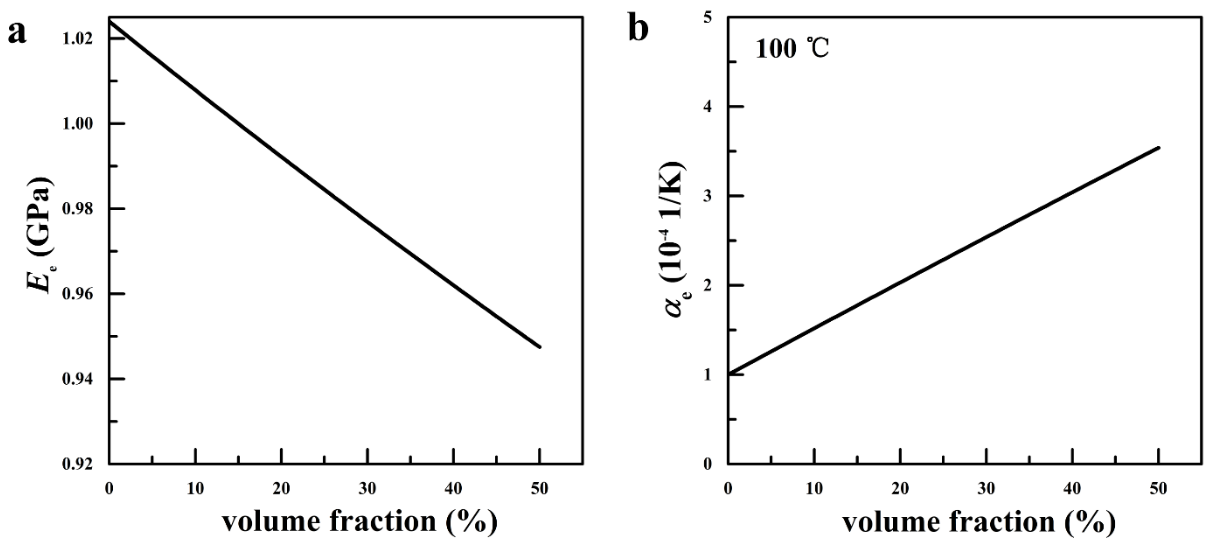

Figure S6. The effective Young's modulus E_e (a) and thermal expansion coefficient α_e at 100 ℃ (b) of the two-layer beam as functions of the droplet volume fraction.

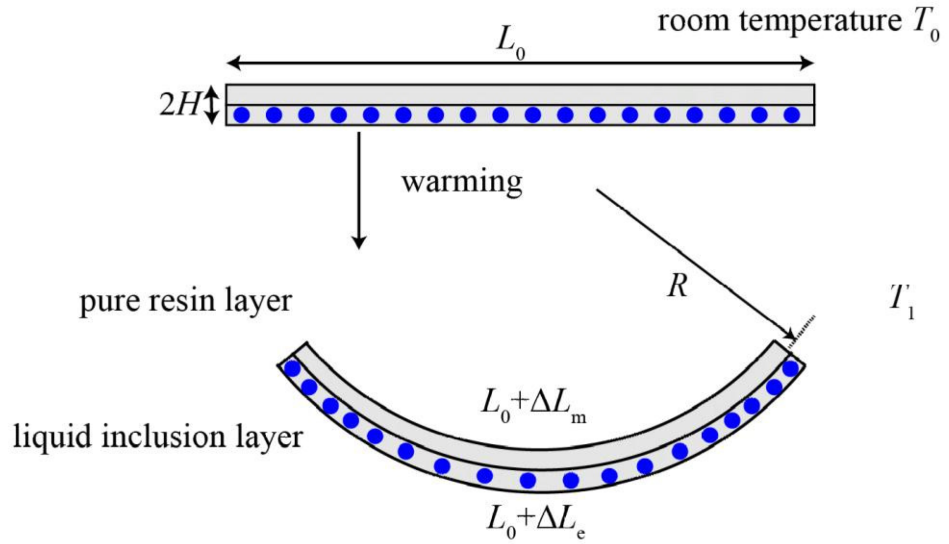

Figure S7. Thermal deflection of the two-layer beam in a temperature rise. The initial straight beam has a length L_0 at the room temperature T_0. An increase in temperature to T_1 induces a larger length increase ΔL_e in the bottom layer than the length increase ΔL_m in the top layer, and the beam becomes curved with a radius of curvature R.
